# Westminster and King's College Hospitals

**Published:** 1918-08-24

**Authors:** 

**Affiliations:** Chairman of the Committee of Management. King's College Hospital, Denmark Hill, S.E.


### Westminster and King's College Hospitals.

THE following letter appeared in the *Times* of August 16. It points to procedure which we have reason to know would not be in accord with the wishes and policy of Sir James Wolfe-Barry, the late chairman of the Westminster Hospital, who did such excellent work and displayed great liberality in his unselfish endeavours to promote its best interests.

THE AMALGAMATION SCHEME.

SIR,—Letters have recently appeared in your columns with reference to a suggestion first made some years ago that a scheme for the amalgamation of Westminster and King's College Hospitals should be considered by the respective governing bodies of these hospitals. So far as my information goes, Sir James Purves Stewart and Mr. Spencer have correctly stated the history of the proposal, but I do not wish at present to follow them into any discussion of its merits. The Committee of Management of this hospital are quite conscious of the difficulties which must be met with in carrying through such a scheme; they realise that the amalgamation of committees and staffs is not a simple matter, but they believe that if all concerned are convinced of the soundness of the proposal difficult details can and will be solved by common sense and good will. Clearly those responsible for the management of Westminster Hospital have a perfect right to their own opinions, but I hope the vice-chairman is alone in his belief that King's College Hospital is anxious for the extinction of Westminster Hospital. No such suggestion has been made or thought of. If amalgamation on principle is agreed to, it must be on a basis of perfect equality as between the two institutions, and the name of each must be preserved.—I am, Sir, yours faithfully,

HAMBLEDEN, Chairman of the Committee of Management.

King's College Hospital, Denmark Hill, S.E.
August 14.

